# Supplementary material for: Subcellular spatial transcriptomics reveals immune–stromal crosstalk within the synovium of patients with juvenile idiopathic arthritis
Source: medRxiv. 2025 Aug 8:2025.08.05.25332835. Preprint. [Version 1] doi: 10.1101/2025.08.05.25332835 (PMC12396606; doi:10.1101/2025.08.05.25332835)
Supplement: Supplement 2 [file media-2.pdf]

Supplementary Table 1: Clinical features of the patients

Patient	JIA 1	JIA 2	JIA 3	JIA 4	JIA 5	JIA 6	JIA 7	JIA 8	JIA 9
**Sex**	F	F	M	F	M	F	F	F	F
**Disease Duration (years)**	0.5	6	3	0.2	0.5	4	0.2	16	0.5
**Time from flare onset to biopsy (years)**	0.1	0.1	0.3	0.2	0.5	0.2	0.2	0.1	0.5
**JIA type**	Oligo	Oligo	Poly	Oligo	Poly	Poly	Oligo	Oligo	Oligo
**Source of Synovium**	Knee	Knee	Knee	Knee	Knee	Knee	Knee	Knee	Knee
**Prior treatment**	Steroid injection	Steroid injection	MTX, LEF, ADA	None	None	Steroid injection, MTX	None	MTX, ETA, ADA	MTX
**Systemic treatment at the time of biopsy**	None	MTX	LEF, ADA	None	None	MTX	None	none	MTX
**Uveitis**	Negative	Negative	Positive	Negative	Negative	Negative	Negative	Positive	Negative
**ANA**	Positive	Positive	Positive	Positive	Positive	Positive	Positive	Negative	Positive
**RF**	Negative	Negative	Negative	Negative	Negative	Negative	Negative	Negative	Negative
**CRP (mg/L)**	0	35	29	11	0	0	0	0	0
**Krenn score (total)**	6	2	5	8	7	6	8	4	6
**Krenn lining**	2	0	1	2	2	2	3	1	2
**Krenn inflammation**	2	1	2	3	3	2	3	1	2
**Krenn stroma**	2	1	2	3	2	2	2	2	2

Oligo: oligoarticular juvenile idiopathic arthritis, Poly: polyarticular juvenile idiopathic arthritis, MTX: methotrexate, LEF: leflunomide, ETA: etanercept, ADA: adalimumab, ANA: antinuclear antibody, RF: rheumatoid factor, CRP: C-reactive protein
